# What can rodent models tell us about apathy and associated neuropsychiatric symptoms in Parkinson's disease?

**DOI:** 10.1038/tp.2016.17

**Published:** 2016-03-08

**Authors:** R Magnard, Y Vachez, C Carcenac, P Krack, O David, M Savasta, S Boulet, S Carnicella

**Affiliations:** 1 Inserm U1216, Grenoble, France; 2 Université Grenoble Alpes, Grenoble Institut des Neurosciences, Grenoble, France; 3 Movement Disorder Unit, Department of Psychiatry and Neurology, CHU de Grenoble, Grenoble, France

## Abstract

In addition to classical motor symptoms, Parkinson's disease (PD) patients display incapacitating neuropsychiatric manifestations, such as apathy, anhedonia, depression and anxiety. These hitherto generally neglected non-motor symptoms, have gained increasing interest in medical and scientific communities over the last decade because of the extent of their negative impact on PD patients' quality of life. Although recent clinical and functional imaging studies have provided useful information, the pathophysiology of apathy and associated affective impairments remains elusive. Our aim in this review is to summarize and discuss recent advances in the development of rodent models of PD-related neuropsychiatric symptoms using neurotoxin lesion-based approaches. The data collected suggest that bilateral and partial lesions of the nigrostriatal system aimed at inducing reliable neuropsychiatric-like deficits while avoiding severe motor impairments that may interfere with behavioral evaluation, is a more selective and efficient strategy than medial forebrain bundle lesions. Moreover, of all the different classes of pharmacological agents, D2/D3 receptor agonists such as pramipexole appear to be the most efficient treatment for the wide range of behavioral deficits induced by dopaminergic lesions. Lesion-based rodent models, therefore, appear to be relevant tools for studying the pathophysiology of the non-motor symptoms of PD. Data accumulated so far confirm the causative role of dopaminergic depletion, especially in the nigrostriatal system, in the development of behavioral impairments related to apathy, depression and anxiety. They also put forward D2/D3 receptors as potential targets for the treatment of such neuropsychiatric symptoms in PD.

In addition to the cardinal motor symptoms of this progressive neurodegenerative disorder, Parkinson's disease (PD) is also associated with a plethora of non-motor manifestations, such as sleep disturbance, cognitive impairment, psychosis, anxiety, depression, apathy and impulsive/compulsive disorders.^1, 2, 3, 4, 5, 6^ This cluster of symptoms, which was largely neglected in the past, and which severely impairs patients' quality of life, is now recognized as a major contributor to morbidity.^2, 3^

Apathy and affective disorders such as depression and anxiety appear as major neuropsychiatric features of the disease.^1, 3, 7^ Apathy, which frequently presents in association with depression and anxiety in PD, is a behavioral syndrome classically defined as a lack of motivation.^7, 8, 9, 10^ Its prevalence in PD varies from 17 to 70% depending on the evaluation scale used and the population studied,^5, 7^ and it is now accepted that a large majority of PD patients will develop apathy along the course of disease.^1, 7, 11, 12^ Apathy is also viewed as a major side effect of deep brain stimulation of the subthalamic nucleus (STN-DBS),^11, 13, 14^ limiting its dramatic motor benefits in terms of patient-related quality of life.^15, 16^ Consequently, understanding the pathophysiology of apathy and associated affective syndromes in PD has gained increasing interest in the medical and scientific communities over the last decade.

After a brief introduction on the phenomenology of apathy and the questions raised by some clinical and imaging studies, we will review and discuss recent advances in the development of rodent models of PD-related neuropsychiatric symptoms. Most of these animal lesion studies point toward the critical role of dopamine in the pathophysiological mechanisms underlying apathy and related affective impairments in PD. They may also provide useful information for the pharmacotherapeutic management of such symptoms.

## Apathy as a core symptom of PD

Although apathetic symptoms were described very early in PD,^17, 18^ awareness of the importance of apathy in the management of PD patients only arose a decade ago. The medical community's lack of interest in this psychiatric impairment was perhaps owing to a lack of nosological clarity. In current psychiatric classification systems, apathy in fact only appears as a feature associated with dementia or with the negative symptoms of schizophrenia, and as a predominant symptom of major depression, overlapping with anhedonia.^5, 19^ More recently, apathy was also referenced as one of the features frequently associated with major or mild neurocognitive disorder caused by Parkinson's or Huntington's disease,^20^ in the same way as anxiety and depression, two affective impairments commonly associated with apathy in these neurodegenerative disorders.^1, 2, 9, 21, 22^ Apathy could therefore be regarded as a nonspecific symptom, stemming from a general degradation of cognitive functions, with negligible implications for clinical evaluation or treatment. However, some evidence suggests that apathy is a true clinical construct,^8, 23^ with interesting transnosographic features.^24, 25, 26^ This led Robert Marin to propose a clear operative definition of apathy and to create the first rating scale in the early 1990s.^8, 27^

Apathy can be defined as deficits in goal-directed behaviors (The term ‘goal-directed behavior' can be misleading and should be viewed as a ‘behavior directed toward a goal' and not as a ‘behavior directed by a goal', as the second has a strong theoretical connotation referring to a specific psychobiological process and putative functional sub-compartmentalization of the dorsal striatum.^28, 29^) or a primary lack of motivation,^27^ characterized by strong deficits in self-initiation and maintenance of voluntary and purposeful behavior, resulting in low levels of activity, loss of socialization and interest in sources of reinforcement.^7, 30, 31, 32^ The observable phenomenon of apathy can be related to emotional, cognitive and auto-activation subdomains, that are underlined by different dysfunctions of the corticostriatal circuits (the so-called basal ganglia limbic, associative and motor loops) involved in the complex chain of processes that transforms an intention into an adapted action.^7, 30^ Therefore, multiple forms of apathy may exist, depending on the respective contribution of each subdomain from one patient to another. Interestingly, a study using an implicit incentive task, revealed that apathy in PD and non-PD patients was associated with an inability to translate an expected reward into effort and action, with no change in the perception of reward value.^31^ The results of this study are in line with recent clinical observations suggesting that the apathetic state described in PD may be particularly linked to the anticipatory subcomponent of anhedonia (which is the absence of any association between pleasure and a specific action), rather than to a change in consummatory responses, which reflect an individual's capacity to experience pleasure when engaged in an enjoyable activity.^33, 34^ Overall, this strongly indicates that the core element of at least some forms of apathy in PD, resides in the motivational preparatory processes responsible for initiating and maintaining voluntary actions.

Apathy appears to be closely linked to anhedonia and complaints of fatigue in PD,^35, 36^ but it is also frequently associated with depression and anxiety. Clinicians have recently regrouped apathy, depression and anxiety into a category called hypodopaminergic behaviors, in opposition to impulsive/compulsive disorders classified as hyperdopaminergic behaviors, to facilitate the clinical management of behavioral complications in PD.^9, 37, 38^ In PD, apathetic symptoms, such as fatigue and lack of interest or initiative, together with depression and anxiety, are often reported even before the onset of motor symptoms, or early in the disease, in *de novo* PD patients.^39, 40, 41, 42^ As already mentioned, apathy is also a major complication of STN-DBS,^11, 13^ particularly in the first postoperative months, when dopaminergic medication has been greatly reduced.^9^ Apathy can also occur later on, with the progression of dysexecutive syndromes related to diffuse cortical spread of alpha-synucleinopathy.^7, 43^ Apathy and associated mood disorders can, moreover, be alleviated at different stages of the disease by dopaminergic treatments, particularly with dopaminergic D2/D3 receptor (D2/D3R) agonists, such as ropinirole, rotigotine, pramipexole or piribedil.^9, 44, 45, 46, 47, 48, 49^ Apathy in PD therefore appears to depend on patient's dopaminergic state, suggesting that dopamine has an important role in the pathophysiology of these non-motor symptoms.^3, 5, 7, 50^ Functional imaging studies in humans have reported an association between PD-related apathy, anxiety and depression, and the extent of dopaminergic denervation in several brain regions, including the ventral and dorsal striatum and the prefrontal cortex (reviewed in ref. 7). Interestingly, similar dopaminergic dysfunctions have been found in the putamen of apathetic Alzheimer disease and Lewy body dementia patients,^51^ thereby strengthening the case for a relationship between apathy and decreased striatal dopaminergic activity.

Despite this useful information, the exact contribution of a dopaminergic deficit to the development of such behavioral impairments in PD, as well as the real therapeutic potential of the different dopaminergic medications, still need to be determined. For instance, it remains unclear whether apathy and associated mood disorders intrinsically derive from the loss of dopamine in the nigrostriatal system or from the diffusion of the lesion towards more putative limbic areas.^7, 30^ Similarly, the aforementioned clinical data clearly suggest that postoperative apathy is the expression of a pronounced hypodopaminergic state revealed by a reduction in dopaminergic medication.^9^ However, STN-DBS may also contribute to the occurrence or aggravation of this syndrome by interfering with the neuronal activity of the non-motor territories of the STN or by stimulating fiber tracts in close vicinity to the STN.^52, 53, 54^ In addition, it has been shown that STN-DBS can interfere directly with dopaminergic function in rats.^55^ Animal models are clearly useful tools to demonstrate dopamine's causal contribution to the pathophysiology of apathy and associated affective disorders in PD. They can also serve to disentangle the respective implication of each potential factor.

## PD-related behavioral impairments in rodent models

Because apathy is operationally defined as a motivational deficit and is frequently associated with depression and anxiety, PubMed and Scopus databases were searched for literature concerning non-motor behaviors in PD-related rodent models (that is, mice and rats) restricted to the evaluation of motivated, anhedonic-like, depression-like or anxiety-like behaviors. References of the publications selected were hand-searched carefully and cross-referenced to find any additional potentially relevant studies. The main information provided by these publications was summarized according to the type of lesional strategy used (Table 1) and to the type of pharmacological treatment used to reverse the behavioral phenotypes induced by the dopaminergic lesions (Table 2). A column was added in Table 1 to indicate the presence or absence of motor impairment, as this may bias interpretation of behavioral results.^56, 57^

## Motivational deficits

### Effects of bilateral and partial dopaminergic lesions of the nigrostriatal pathway

Evaluating operant behaviors in rodents appears particularly relevant for the study of the psychobiological mechanisms underlying apathy, because it allows a fine and sophisticated evaluation of motivational preparatory processes in both human and non-human species (for review, see ref. 78). Partial dopaminergic denervation, limited to the nigrostriatal system (<80% tyrosine hydroxylase immunoreactivity loss within the dorsal striatum and <20% tyrosine hydroxylase immunoreactivity loss within the nucleus accumbens) and induced by the bilateral stereotaxic infusion of the catecholaminergic neurotoxin 6-hydroxydopamine (6-OHDA) into the substantia nigra pars compacta (SNc), dramatically impaired operant behaviors in rats (Table 1).^56, 61, 62^ Specifically, diminished performance in a runway task involving getting to and eating palatable food, and a significant reduction in instrumental response to sucrose in an operant self-administrating procedure were observed in 6-OHDA-SNc-lesioned rats.^56^ Importantly, because the partial SNc lesion altered neither sensorimotor coordination on a rotarod nor fine-motor velocity in an automated laboratory gait analysis system, these deficits cannot be attributed to motor impairments.^56^ Moreover, the ability of lesioned animals to press an operant lever at short intervals was preserved.^61^ Similarly, these results cannot be attributed to an alteration of the rewarding/reinforcing properties of the reinforcers because neither conditioned place preference for the palatable food used in the runway, nor preference for sucrose in a two-bottle choice procedure, were impaired by the dopaminergic lesion.^56^ Therefore, a marked motivational deficit was observed specifically when an instrumental preparatory action was required, a behavioral phenotype that appears highly reminiscent of at least some forms of apathy.^7, 30, 31^

These data also corroborate the observation that severe, unilateral dopaminergic depletion, targeting, but not restricted to, the dorsal striatum or bilateral dopaminergic depletion restricted to the dorsal striatum, reduced operant performances of rats in a choice reaction time task.^79, 80^ Rats with bilateral partial SNc lesions induced by 6-OHDA also exhibited increased latency to complete the 100-pellet test.^81^ Although consummatory or fine-motor deficits may contribute to these results,^81^ overall, the aforementioned data^56, 79^ suggest that increased latency in reaching and eating a large number of food reward pellets, which requires, as in the runway task, a high degree of behavioral activation,^82^ reflects a real decrease in motivation.

### Effects of bilateral and partial dopaminergic lesions of the mesolimbic pathway

Some studies suggest that apathy and associated neuropsychiatric symptoms in PD may stem from dysfunctions of the dopaminergic mesocorticolimbic system.^9, 10^ Partial and bilateral 6-OHDA lesions affecting the medial ventral tegmental area (medial VTA) were therefore also performed (Table 1 and ref. 56) in order to mimic the partial dopaminergic denervation of the ventral striatum that frequently occurs in PD patients, especially in the late stage of the disease.^83, 84^ Interestingly, none of the motivational deficits described in the section ‘Effects of bilateral and partial dopaminergic lesions of the nigrostriatal pathway' above were observed with a 60–70% loss of dopaminergic innervation in the nucleus accumbens^56^ (see also ref. 81). As the mesolimbic dopaminergic system is a key player in reward-related and goal-directed behaviors,^85, 86^ this absence of effect may appear counterintuitive. However, as we have known even since the early days of 6-OHDA use,^87^ a complete, or near complete, loss of dopamine in the mesolimbic pathway is necessary to obtain a reduction in motivated behavior (for review, see refs 88, 89). Thus, this striking dissociation between the behavioral effects of a partial dopaminergic lesion of the VTA or the SNc clearly strengthens the case for the involvement of the nigrostriatal system in the pathophysiology of apathy in PD on top of the mesolimbic and mesocortical projection systems.

### Pharmacological treatments

To further investigate the role of dopamine, different dopaminergic agents classically used in PD were tested on the motivational deficits induced by a partial and bilateral nigrostriatal lesion (Table 2). The D2/D3R agonists ropinirole and pramipexole, efficiently corrected the decrease in operant sucrose self-administration induced by the SNc lesions, whereas the dopamine precursor l-DOPA did not.^56, 61^ Moreover, this deficit was not reversed by subchronic administration of the selective serotonin reuptake inhibitor (SSRI) citalopram. Using more selective dopaminergic receptor subtype agonists, the motivational deficits induced by the SNc lesions were specifically reversed by the D3R agonist PD-128907, but not by the D1R agonists SKF-38393 and SKF-82958, nor by the D2R agonist sumanirole.^62^ Taken together, these data confirm the implication of dopamine and point toward the targeting of D3R for reversing the motivational deficits related to PD.

## Depression-like behaviors

### Effects of bilateral and partial dopaminergic lesions of the nigrostriatal pathway

In keeping with the high incidence of depressive symptoms in PD patients, most of the studies found that partial and bilateral dopaminergic denervation of the nigrostriatal pathway induces a depression-like phenotype (Table 1). Indeed, anterograde and retrograde dopaminergic lesions of the dorsal striatum increased immobility, a classical behavioral index of depression-like state,^90^ in the forced-swim test (FST) as well as in the tail-suspension test, in rats and mice, respectively. Importantly, motor impairments cannot account for a reduction in swimming or climbing, as no major motor deficits were identified with these partial-lesion approaches.^56, 65, 66, 68^

### Effects of bilateral and partial dopaminergic lesions of the mesolimbic pathway

Although the mesolimbic dopaminergic system has been shown to be involved in the regulation of mood and depression-related behaviors,^91, 92^ partial dopaminergic lesion of the VTA did not increase the time that rats spent immobile in a FST.^56^ However, there is a lack of data and more studies are needed to determine the potential impact of partial dopaminergic denervation of the mesolimbic pathway on such behaviors.

### Effects of MPTP and 6-OHDA medial forebrain bundle lesions

Studies on mice using systemic administration of MPTP (1-methyl-4-phenyl-1,2,3,6-tetrahydropyridine) toxin, and on rats using unilateral or bilateral 6-OHDA medial forebrain bundle (MFB) lesion usually result in extensive and non-selective dopaminergic lesions affecting both the nigrostriatal and mesolimbic pathways.^69, 71^ As shown in Table 1, such lesion-based strategies have yielded conflicting results: some studies showed increased immobility in the tail-suspension test^60^ or increased latency in terminating foot shock presentation during a learned helplessness test,^69^ whereas others did not observe any depression-like behaviors in rodents,^72, 77^ even in the case of bilateral 6-OHDA-MFB lesions.^75^ The absence of lesional selectivity and the presence of motor impairments in these studies are likely to account for these conflicting results, thereby revealing a strong limitation of MFB and MPTP lesions in the study of non-motor behaviors in rodents.

### Pharmacological treatments

Regarding motivational deficits, dopaminergic agents were tested to reverse depression-like phenotypes in rodent models of PD (Table 2). Subchronic administration of l-DOPA has been shown to correct the increase in immobility of lesioned rats in the FST^56^ and to reduce latency in terminating the foot shock in a learned helplessness procedure.^69^ However, other studies failed to show any beneficial effect of l-DOPA on depression-like behaviors in rats^70, 75^ or mice.^58^ By contrast, D2/D3R agonists such as pramipexole, consistently improved depression-like behaviors, whatever the lesional or animal model used. Similar positive results were obtained with selective D1R, D2R or D3R agonists.^62^

Surprisingly, inconsistent effects were found with SSRIs (Table 2) and these mixed results may be a product of the behavioral tests and/or the lesional model used. For instance, citalopram reduced depression-like behaviors in unilateral MFB-lesioned rats in a learned helplessness procedure,^69^ but not in bilateral SNc-lesioned rats in the FST.^56^ Fluoxetine significantly decreased FST-immobility in controls but not in rats with retrograde dopaminergic lesions of the dorsal striatum.^67^ Finally, the serotonin/noradrelanine reuptake inhibitor imipramine failed to improve depression-like behaviors in the same study,^67^ whereas the noradrenaline reuptake inhibitor reboxetine succeeded in 6-OHDA-lesioned mice,^58^ as did sarizotan, a serotoninergic and dopaminergic partial receptor agonist, in unilateral MFB-lesioned rats.^73^ It should also be highlighted that these studies also differ in terms of treatment duration, which is a critical factor to obtain efficient antidepressant actions using SSRI. Indeed, the same dose of fluoxetine has been found to significantly reduce depression-like behaviors after 21 (ref. 93) but not 13 (ref. 67) days of subchronic administration. More consideration should be given to this methodological issue in the future.

Overall, these data strongly indicate the predominant involvement of dopamine, and of the other monoaminergic systems, in parkinsonian-related depression-like behaviors, and show that the dopaminergic receptors are potential therapeutic targets for the treatment of depression in PD.

## Anhedonia-like behaviors

### Lesional effects

Hedonic processes can be divided into consummatory and anticipatory subcomponents, at least from a psychological and neurobiological point of view.^33, 34^ In this part of the review, we will use the term anhedonia to refer only to deficits in reward-related processes accompanied by a failure to experience pleasure leading to a reduction in consummatory behavior (that is, the equivalent of ‘liking' deficits, as termed by Berridge^85^). However, because subjective feelings such as pleasure remain quite difficult to apprehend in rodents, from an operational point of view, we refer to anhedonia in preclinical studies as a behavioral insensitivity to the rewarding or reinforcing properties of a specific substance or event.

All the studies summarized in Table 1 used two-bottle choice procedures with water and sucrose as the rewarding solution to assess anhedonia. No clear results emerged from this literature, irrespective of the type of lesional strategy used, which may be due to behavioral protocol differences in these studies and due to the difficulty in isolating and accurately assessing this psychobiological subcomponent of motivated behaviors. Some studies used food and water deprivation before testing,^60, 64, 65, 71^ or short 1 h (ref. 68) versus standard 24 h access to the sucrose solution, and this may have interfered with the task by influencing motivational processes. Moreover, all the studies did not use the same sucrose concentration. For instance, partial and bilateral dopaminergic lesions of the nigrostriatal pathway have been found to reduce preference for a 0.5 or a 0.8%, but not for a 2% sucrose solution.^56, 66, 68^ When preference was reduced, it was also unclear whether this resulted from a decrease in sensitivity to the rewarding properties of sucrose or from a metabolic confounding factor because lesions of this type can interfere dramatically with hypothalamic function.^94^ In order to exclude this potential bias, one study replaced sucrose with saccharin and found that preference for this non-caloric sweetener was preserved in SNc-lesioned rats.^56^

A growing body of evidence suggests that dopamine is more involved in motivational (‘wanting' or anticipatory/preparatory) behaviors than in rewarding or hedonic processes *per se* (‘liking' or consummatory behaviors).^85, 95^ These studies do not, therefore, make it clear whether or not dopaminergic lesions really can induce anhedonia or emotional apathy in humans.

### Pharmacological treatments

Very few drugs have been tested on these types of behavior (Table 2). However, results suggest a specific involvement of dopamine, as l-DOPA^71^ and apamin, a SK channel blocker originally isolated from bee venom known to stimulate the dopaminergic function, improved anhedonia-like behaviors,^68^ while the SSRI paroxetine and the noradrenaline/dopamine reuptake inhibitor bupropion failed to do so.^71^

## Anxiety-like behaviors

### Lesional effects

As is the case for depression-like behaviors, partial and bilateral dopaminergic denervation of the nigrostriatal pathway consistently induced anxiety-related behaviors (Table 1, but see ref. 65). Anterograde and retrograde dopaminergic lesions of the dorsal striatum reduced the amount of time spent in the open arms of an elevated plus maze, a classical index of anxiety. Reduced latency to enter into the dark side of a light/dark avoidance apparatus was also observed in SNc-lesioned rats.^56^ Moreover, 6-OHDA SNc-lesioned mice exhibited a pronounced increase in thigmotaxis, as indicated by a reduction in time spent in the center of the open-field apparatus.^58^ Again, no consistent results were obtained from studies using 6-OHDA MFB lesions (Table 1).

Interestingly, bilateral and partial lesion of the VTA did not induce an anxiety-like phenotype, either in the elevated plus maze^56, 76^ or in the light/dark avoidance test,^56^ thereby strengthening the implication of the dopaminergic nigrostriatal system in the regulation of affective and emotional functions.

### Pharmacological treatments

In keeping with the deleterious impact of nigrostriatal dopaminergic denervation on mood, anxiety-related behaviors in lesioned animals have been shown to be responsive to dopaminergic medication, especially to dopaminergic receptors agonists (Table 2). For instance, anxiety-like behaviors in lesioned mice in the elevated plus maze were reversed by pramipexole but not by l-DOPA,^58^ suggesting a greater efficacy of direct receptor agonists. Interestingly enough, while some agonists such as ropinirole, pramipexole or the D3R agonist PD-128907 normalized anxiety-related behaviors to the level of control animals,^56, 58, 62^ the D1R agonist SKF-38393 and the D2R agonist sumanirole produced striking anxiolytic effects on lesioned rats.^62^ Depending on the dose used, SKF-38393 and sumanirole could increase the time spent in the open arms of the elevated plus maze, in SNc-lesioned rats specifically, to levels far above those obtained in their respective control conditions, and promoted disinhibition and risk-taking behaviors, as reflected by an increased number of head dippings and reduction of risk assessments.^62^ Although these data deserve further investigation, they already provide useful information about the iatrogenic mechanisms of dopaminergic medications that may lead to the development of impulsive/compulsive disorders in PD.

In addition to the dopaminergic system, preclinical studies suggest that targeting the other monoaminergic systems may also be a valuable strategy for the treatment of anxiety in PD. For instance, systemic administration of serotoninergic or noradrenergic reuptake inhibitors efficiently improved anxiety-related behaviors in lesioned animals^56, 58^ (but see refs 67). Similar beneficial effects were obtained with intra-amygdala or -prelimbic infusion of the 5-HT1A receptors agonist 8-OH-DPAT.^74, 96^

## Conclusions and future directions

Models based on 6-OHDA-lesioned rats constitute the most widely used and best-characterized experimental approach for PD.^97^ Initially developed to study motor symptoms (for review, see ref. 97), all 6-OHDA models are not equivalent and relevant when it comes to mimic and examine the neuropsychiatric symptoms observed in PD, that is, apathy, depression, anhedonia and anxiety. The recent studies reviewed here tend to show that a selective, partial and bilateral lesion of the nigrostriatal dopaminergic pathway (with a retrograde or an anterograde strategy) is probably the most suitable approach in the investigation of the neurobehavioral mechanisms that underlie neuropsychiatric symptoms in PD using 6-OHDA. There is now a large body of evidence highlighting the critical role of the dopaminergic nigrostriatal system—the major neuronal group known to degenerate in PD—in motivational, affective and cognitive impairments. Although this role was previously largely neglected and mainly attributed to the dopaminergic mesoaccumbal pathway,^98^ these data are consistent with pioneering studies supporting a role of nigrostriatal dopamine in motivation^99, 100, 101, 102^ or with more recent evidence based on electrophysiological recordings in monkeys^103, 104^ (for review, see ref. 86) and selective optogenetic modulation of nigral dopaminergic neurons.^105, 106^ Furthermore, partial and selective lesion of this nigrostriatal pathway allows the circumvention of motor symptoms, a major bias in the study of non-motor symptoms.^57^ Such an advantage is not found in more widespread lesional models such as MFB lesion.^60, 64, 69, 71, 72^

In addition, the relevance of rodent models based on partial and selective degeneration of the nigrostriatal pathway is strengthened by their important predictive value concerning the pharmacological treatment used to reverse neuropsychiatric symptoms in clinic. Interestingly, the pharmacological studies summarized in this review show that medications which produce positive results on motivational deficits are also effective on depression- and anxiety-like behaviors, and on anhedonia-like behaviors, potentially indicating some common mechanisms between these neuropsychiatric symptoms in PD.^56, 59, 61, 62, 68, 69, 71^ Even if these mechanisms remain elusive, these data, overall, highlight the major implication of dopamine and clearly indicate that dopaminergic receptors constitute promising therapeutic targets. On this last point, the literature presented here suggests that direct agonists such as pramipexole or ropinirole may be more efficient to reduce neuropsychiatric symptoms of PD than a more global modulation of dopaminergic neurotransmission with l-DOPA. More precisely, it indicates that pharmacological interventions on D3R are especially appropriate for the reversal of these deficits, as can be seen from the fact that the D3R-prefering PD-128907 reversed motivational deficits in PD models, whereas the D2R selective agonist sumanirole did not (Table 2). Importantly, the D3R is suspected to be a key player in the control of affective and motivated behaviors and as such, has been identified as a potential target for the treatment of neuropsychiatric symptoms in several disorders.^107, 108^ D3R expression is particularly high within the ventral striatum, but is also detectable in the dorsal striatum,^109, 110, 111^ where it has been shown to significantly participate in the control of motivated behaviors.^111, 112^ In accordance with the existing overlap between VTA and SNc dopaminergic projections^113^ and the emerging role of the dorsal striatum in non-motor behaviors (for review, see also refs 86), this distribution challenges the oversimplified vision of perfectly segregated systems which serve specific functions (that is, limbic, associative and motor). This appears even more relevant in PD, where dopaminergic denervation in combination with dopaminergic treatments can lead to complex and widespread postsynaptic redistribution of D3R, with notably a strong increase of its expression in the dorsal striatum.^109, 114^ Because of this interesting predictive and potential face and construct validity, these rodent models could also serve to determine the relative psychotropic potencies of the different dopaminergic medications.^115^ It corresponds to a clinical need,^116^ namely to avoid a potential source of hypo- and hyperdopaminergic psychiatric side effects in the management of PD, as l-dopa equivalent dosages of different dopamine agonists are only known for motor effects.

Some studies in non-human primates also confirm a major involvement of dopamine in the pathophysiology of apathy in PD. For instance, MPTP lesions in monkey can lead to a decrease in behavioral activity^117^ and goal-directed behaviors,^118, 119^ and to social behavioral changes.^120^ However, because of the lack of neuroanatomical selectivity of systemic MPTP administration, and despite in-depth multi-correlational and neuroimaging analyses, it has yet to be determined from these studies whether these behavioral impairments result from a loss of dorso- or ventro-striatal function.

Although the dopaminergic system seems to have a crucial role in the physiopathology of neuropsychiatric symptoms in PD, it does not exclude the implication of the other monoaminergic systems, particularly when the strength of their interconnections is taken into consideration. The noradrenergic and serotoninergic systems are also affected in PD^72^ and some of the neurochemical,^66^ behavioral and pharmacological studies^56, 58, 69, 72, 121^ reviewed here support their potential implication in non-motor impairments. Affective-related impairments in lesioned animals can indeed be reduced by SSRIs or noradrenergic reuptake inhibitors (Table 2). Moreover, affective-related behaviors in rats with unilateral dopaminergic lesions may emerge only in combination with serotoninergic and/or noradrenergic depletion^72^ and the degree of depression-like behaviors in 6-OHDA-lesioned rats has been recently shown to correlate with hippocampal and striatal serotoninergic dysfunctions.^63, 122^ Functional imaging studies in humans and other clinical evidence also suggest that depression and fatigue in PD are associated with alterations of the serotonergic system (reviewed in ref. 123), and, in addition to dopamine, anxiety, depression and apathy in PD patients may also be associated with a greater loss of noradrenaline in the locus coeruleus and noradrenergic innervation in the ventral striatum and other limbic areas.^10^ Although more studies are needed to decipher the specific role of these different neurotransmitter systems, it might indicate that PD ought to be considered as a monoaminergic pathology. Combining pharmacotherapies, to target the three monoaminergic systems, may therefore be a particularly valuable strategy in the treatment of neuropsychiatric symptoms related to the neurodegenerative process in PD.

Taken together, the preclinical results reviewed here clearly favor the hypothesis that postoperative apathy rather results from the reduction of the dopaminergic medication following DBS - thereby revealing a strong hypodopaminergic state related to non-motor functions - than by STN-DBS *per se*. How dopaminergic denervation of a putative motor system interferes so strongly with motivated and affective-related behaviors remains, however, unclear. As already aforementioned, the three so-called limbic, associative and motor cortico-striato-cortical loops are not fully segregated, with an ascending striato-midbrain-striatal spiraling circuitry,^28, 124, 125, 126^ that functionally interconnects the different corticostriatal regions and associated structures,^127, 128^ and a high degree of overlap and convergence between these functional territories within the STN.^14, 129^ One can therefore speculate that loss of nigrostriatal dopamine might influence non-motor functions through these subcortical interactions, thereby disrupting the complex chain of events that bridges emotions to actions. A similar scenario can be envisaged within the framework of the actor-critic model of the basal ganglia,^130, 131^ with a functional disconnection of the actor (the dorsal striatum) and the critic (the ventral striatum). Depending on the studies, it is suggested that STN-DBS can induce either hypomania and euphoria^132, 133, 134, 135^ or apathy.^52, 53^ The STN is a small nucleus quite difficult to target and differences in electrode localizations may partly account for these discrepancies. Specifically, induction of hypomania may be associated with stimulation of ventral, that is, limbic, parts of the STN,^134, 135^ whereas inaccurate electrode placements or exaggerated current wide-spreading to surrounding areas, such as the substantia nigra *pars reticulata,*^136^ or pallidothalamic fiber tracts^54^ may exert opposite actions. It may, therefore, be of great interest to investigate how the dopaminergic lesion and DBS may interact agonistically or antagonistically, by dissecting the effects of STN-DBS on the three cortico-striato-cortical loops using, for example, optogenetics tools.^137^

In conclusion, toxin-induced lesions of the nigrostriatal DAergic neurons that have been used to model PD in rodents since the 1960s have recently been re-evaluated for their ability to model some non-motor symptoms and neuropsychiatric symptoms in particular. Thus, efforts have begun to shift away from models that induce destruction of the vast majority of the DA cells of the SNc to more gradual and specific models that allow for the analysis of non-motor symptoms. Nevertheless, in the future, the study of non-motor symptoms in PD will need to be extended to recently developed genetic- or AAV α-synuclein-based rodent models, as interesting complementary approaches to study the occurrence of neuropsychiatric symptoms throughout the progressive neurodegenerative process characteristic of PD. Viral vector-induced overexpression of wild-type human α-synuclein in nigral dopaminergic neurons of rats, for instance, induced a depression-like phenotype.^138^ Transgenic mice expressing human α-synuclein also showed early and severe olfactory deficits,^139^ reproducing one of the most prominent non-motor, and premotor, symptoms of PD.^2, 3^ These studies highlight how relevant genetic and viral approaches are in the quest to unravel the mechanisms underlying neuropsychiatric symptoms in PD, to screen drugs and to test the efficiency, or to reveal potential side effects, of neurosurgical approaches such as DBS on such non-motor symptoms.^64, 140, 141^

## Figures and Tables

**Table 1:** Animal lesional models for neuropsychiatric phenotypes of Parkinson's disease

*Species/strain*	*Toxin*	*Hemisphere*	*Injection site*	*Lesional extent*		*Phenotypes*					*References*
				*Striatum*	*Midbrain*	*Motivational deficits*	*Depression-like*	*Anhedonic-like*	*Anxiety-like*	*Motor deficits*	
*Mouse*											
C57BL/6 J	6-OHDA	Bilateral	DLS	65 to 75% TH-IR loss striatum	Not given	NT	+	NT	+	±	Bonito-Oliva *et al.*^58, 59^
C57BL/6 J	MPTP	Bilateral	i.p.	45% TH-IR DS	66% TH-IR loss SNc	NT	+	−	±	(NP) −	Vuckovic *et al.*^60^
C57BL/6 J	MPTP	Bilateral	i.p.	90–95% loss striatal DA (HPLC)	60–70% loss SNc neurons	NT	−	−	+	+	Gorton *et al.*, 2010
											
*Rat*											
Sprague Dawley	6-OHDA	Bilateral	SNc	<80% TH-IR loss DS and <20% TH-IR loss NAc	<95% TH-IR loss SNc and <35% TH-IR loss VTA	+	NT	NT	NT	−	Favier *et al.*^61^
Sprague Dawley	6-OHDA	Bilateral	SNc	<80% TH-IR loss DS and <15% TH-IR loss NAc	Not given	+	+	NT	+	NT	Carnicella *et al.*^62^
Sprague Dawley	6-OHDA	Bilateral	SNc	<70% loss TH-IR DS and <5% TH-IR loss NAc	<75% loss TH-IR SNc and <5% TH-IR loss mVTA	+	+	−	+	−	Drui *et al.*^56^
Wistar	6-OHDA	Bilateral	SNc	Not given	<30% TH-IR loss SNc	NT	+	+	NT	−	Santiago *et al.*^63^
Wistar	6-OHDA	Bilateral	SNc	Not given	<45% TH-IR loss SNc	NT	−	−	+	+	Campos *et al.*^64^
Wistar	6-OHDA	Bilateral	DLS	35% loss striatal DA (HPLC)	Not given	NT	+	(NP) −	−	−	Branchi *et al.*^65^
Wistar	6-OHDA	Bilateral	Ventrolateral area of DS	<50% TH-IR loss striatum	<30% TH-IR loss SNc	NT	+	+	+	−	Tadaiesky *et al.*^66^
Wistar	6-OHDA	Biateral	Ventrolateral region of DS	Not given	25% TH-IR loss SNc and 20% TH-IR loss VTA	NT	+	NT	NT	−	Berghauzen-Maciezjeska *et al.*^67^
Wistar	6-OHDA	Bilateral	DS	<60% striatum TH-IR loss	Not given	NT	NT	+	+	−	Chen *et al.*^68^
Wistar	6-OHDA	Left	Striatum	Not given	45%, 75%, 100% TH-IR loss SNc and 30%, 50% TH-IR loss VTA	NT	+	NT	NT	−	Winter *et al.*^69^
Wistar	6-OHDA	Left	MFB			NT	+	NT	NT	−	Winter *et al.*^69^
Sprague Dawley	6-OHDA	Left	MFB	100% loss striatal DA (HPLC)	Not given	NT	−	NT	+	+	Jaunarajs *et al.*^70^
Wistar Han	6-OHDA	Right	MFB	Not given	75% SNpc and 40% VTA TH-IR loss 100% SNpc and 60% VTA TH-IR loss	NT	NT	+	−	+	Carvalho *et al.*^71^
Wistar	6-OHDA	Right	MFB	95% loss striatal DA (HPLC)	Not given	NT	−	−	−	+	Delaville *et al.*^72^
Sprague Dawley	6-OHDA	Right	MFB	<100% TH-IR loss striatum	Not given	NT	+	NT	+	−	Zhang *et al.*^73^
Sprague Dawley	6-OHDA	Right	MFB	Not given	100% TH-IR loss SNc and 35% TH-IR loss VTA	NT	NT	NT	+	+	Sun *et al.*^96^
Sprague Dawley	6-OHDA	Bilateral	MFB	Not given	50% TH-IR loss SNpc	NT	−	NT	−	+	Jaunarajs *et al.*^75^
Sprague Dawley	6-OHDA	Bilateral	mVTA	<70% loss TH-IR NAc and <25% TH-IR loss DS	<40% loss TH-IR mVTA and <10% TH-IR loss SNc	−	−	−	−	−	Drui *et al.*^56^
Sprague Dawley	6-OHDA	Bilateral	VTA	Not given	35% TH-IR loss VTA	NT	NT	+	−	−	Dardou *et al.*^76^

Abbreviations: +, positive result for the test; ±, positive trend for the test; −, negative result for the test; 6-OHDA, 6-hydroxydopamine; aVTA, anterior ventral tegmental area; DA, dopamine; DLS, dorsolateral striatum; DS, dorsal striatum; HPLC, high pressure liquid chromatography; i.p., intraperitoneal; MFB, medial forebrain bundle; MPTP, 1-methyl-4-phenyl-1,2,3,6-tetrahydropyridine; mVTA, medial ventral tegmental area; NAc, nucleus accumbens; NP, not presented; NT, not tested; pVTA, posterior ventral tegmental area; SNc, substantia nigra pars compacta; TH-IR, tyrosine hydroxylase immunoreactivity.

**Table 2:** Treatment tested on animal lesional models for neuropsychiatric phenotypes of Parkinson's disease

*Compound*	*Mechanism*	*Dose*	*Treatment duration*	*Phenotypes*				*References*
				*Motivational deficit*	*Depression-like*	*Anhedonic-like*	*Anxiety-like*	
l-DOPA*	DA precursor	10 mg kg^−1^; i.p.	4 days	NT	−	NT	−	Bonito-Oliva *et al.*^58^
l-DOPA*	DA precursor	10 mg kg^−1^; i.p.	Not given	NT	NT	NT	+	Zhang *et al.*^73^
l-DOPA^+^	DA precursor	12 mg kg^−1^; s.c.	75 days	NT	−	NT	−	Jaunarajs *et al.*^75^
l-DOPA^+^	DA precursor	12 mg kg^−1^; s.c.	28 days	NT	−	NT	−	Jaunarajs *et al.*^77^
l-DOPA^+^	DA precursor	12,5 mg kg^−1^; i.p.	3 to 14 days	−	+	NT	+	Drui *et al.*^56^
l-DOPA°	DA precursor	25 mg kg^−1^; i.p.	3 days	NT	+	NT	NT	Winter *et al.*^69^
l-DOPA^-^	DA precursor	25 mg kg^−1^; oral	Not given	NT	NT	+	NT	Carvalho *et al.*^71^
SKF-38393	D1R agonist	2.5 or 3.5 mg kg^−1^; i.p.	3 to 14 days	−	+	NT	+	Carnicella *et al.*^62^
Sumarinole	D2R agonist	0.1 or 0.15 mg kg^−1^; i.p.	3 to 14 days	−	±	NT	+	Carnicella *et al.*^62^
Ropinirole	D2/D3R agonist	1 mg kg^−1^; i.p.	3 to 14 days	+	+	NT	+	Drui *et al.*^56^
Pramipexole	D2/D3R agonist	0.1 mg kg^−1^; s.c.	32 days	NT	NT	+	−	Dardou *et al.*^76^
Pramipexole	D2/D3R agonist	0.2 mg kg^−1^; i.p.	12 days	+	NT	NT	NT	Favier *et al.*^61^
Pramipexole	D2/D3R agonist	0.6 mg kg^−1^; s.c.	4 days	NT	+	NT	+	Bonito-Oliva *et al.*^59^
Pramipexole	D2/D3R agonist	1 mg kg^−1^; s.c.	13 days	NT	+	NT	−	Berghauzen-Maciezjeska *et al.*^67^
PD-128907	D3R agonist	0.1 or 0.15 mg kg^−1^; i.p.	3 to 14 days	+	+	NT	+	Carnicella *et al.*^62^
Paroxetine	SSRI	6 mg kg^−1^; i.p.	Not given	NT	NT	−	NT	Carvalho *et al.*^71^
Citalopram	SSRI	10 mg kg^−1^; i.p.	3 to 14 days	−	−	NT	+	Drui *et al.*^56^
Fluoxetine	SSRI	10 mg kg^−1^; i.p.	13 days	NT	±	NT	−	Berghauzen-Maciezjeska *et al.*^67^
Citalopram	SSRI	10 mg kg^−1^; i.p.	3 days	NT	+	NT	NT	Winter *et al.*^69^
Sarizotan	5-HT1AR agonist and D2R-like partial agonist	1 mg kg^−1^	Not given	NT	+	NT	NT	Zhang *et al.*^73^
8-OH-DPAT	5-HT1AR agonist	0.5 μg intra CeM	Acute	NT	NT	NT	+	Sun *et al.*^96^
Imipramine	5-HT/ NA reuptake inhibitor	10 mg kg^−1^; i.p.	13 days	NT	−	NT	−	Berghauzen-Maciezjeska *et al.*^67^
Reboxetine	NA reuptake inhibitor	20 mg kg^−1^; i.p.	4 days	NT	+	NT	+	Bonito-Oliva *et al.*^59^
Bupropion	NA/ DA reuptake inhibitor	10 mg kg^−1^; i.p.	Not given	NT	NT	−	NT	Carvalho *et al.*^71^
Apamin	Sk channel blocker	0.1 or 0.3 mg kg^−1^; i.p.	11 days	NT	NT	+	+	Chen *et al.*^68^

Abbreviations: +, positive result for the test; −, negative result for the test; 5-HT, 5-hydroxytryptamine; CeM, medial subdivision of the central nucleus of amygdala; D1/D2/D3R, dopaminergic receptor 1, 2, 3; DA, dopamine; DMS, dorsomedial striatum; i.p., intraperitoneal; l-DOPA, l-3,4-dihydroxyphenyl-l-alanine; NA, noradrenaline; Not given, data was not clearly developed in the mentioned study; NT, not tested; s.c., subcutaneous; SSRI, selective serotonin reuptake inhibitor. Superscripts to l-DOPA: *, ^+^, ° indicate treatment respectively combined with 7.5 mg kg^−1^, 15 mg kg^−1^ and 6.25 mg kg^−1^ of peripheral DOPA decarboxylase inhibitor benserazide; ^−^ indicates treatment combined with 5 mg kg^−1^ of DOPA decarboxylase inhibitor carbidopa.
